# Effect of Genetic Polymorphisms and Long-Term Tobacco Exposure on the Risk of Breast Cancer

**DOI:** 10.3390/ijms17101726

**Published:** 2016-10-14

**Authors:** Zoraida Verde, Catalina Santiago, Luis Miguel Chicharro, Luis Reinoso-Barbero, Alejandro Tejerina, Fernando Bandrés, Félix Gómez-Gallego

**Affiliations:** 1 Department of Biomedical Sciences, European University, Madrid 28670, Spain; luis.reinoso@universidadeuropea.es; 2 School of Doctoral Studies & Research, European University, Madrid 28670, Spain; catalina.santiago@universidadeuropea.es (C.S.); felix.gomez@universidadeuropea.es (F.G.-G.); 3 Cátedra Complutense Diagnostic and Innovation, Universidad Complutense, Madrid 28040, Spain; luismi.chicharro@gmail.com (L.M.C.); fbandres@med.ucm.es (F.B.); 4 Centro de Patología de la Mama-Fundación Tejerina, Madrid 28003, Spain; alejandro.tejerina@fjd.es; 5 Department of Toxicology and Health Sanitary, Complutense University of Madrid, Madrid 28040, Spain

**Keywords:** breast cancer, long-term smoking, CYP1A, DNA repair genes

## Abstract

Introduction: Tobacco smoke contains many potentially harmful compounds that may act differently and at different stages in breast cancer development. The focus of this work was to assess the possible role of cigarette smoking (status, dose, duration or age at initiation) and polymorphisms in genes coding for enzymes involved in tobacco carcinogen metabolism (*CYP1A1*, *CYP2A6*) or in DNA repair (*XRCC1*, *APEX1*, *XRCC3* and *XPD*) in breast cancer development. Methods: We designed a case control study with 297 patients, 217 histologically verified breast cancers (141 smokers and 76 non-smokers) and 80 healthy smokers in a cohort of Spanish women. Results: We found an association between smoking status and early age at diagnosis of breast cancer. Among smokers, invasive carcinoma subtype incidence increased with intensity and duration of smoking (all *P*_trend_ < 0.05). When smokers were stratified by smoking duration, we only observed differences in long-term smokers, and the *CYP1A1 Ile462Ile* genotype was associated with increased risk of breast cancer (OR = 7.12 (1.98–25.59)). Conclusions: Our results support the main effect of CYP1A1 in estrogenic metabolism rather than in tobacco carcinogen activation in breast cancer patients and also confirmed the hypothesis that *CYP1A1 Ile462Val*, in association with long periods of active smoking, could be a breast cancer risk factor.

## 1. Introduction

Tobacco smoking is among the leading preventable risk factors for variety of diseases [1]. Evidence regarding active cigarette smoking and breast cancer risk remains inconclusive. Several compounds in tobacco smoke, such as polycyclic hydrocarbons, aromatic amines and *N*-nitrosamines may induce breast tumours [2,3].

In Europe, breast cancer is the third most common oncological disease cause of death. In addition, breast cancer is the most frequent type of cancer in women younger than 50 years old [4]. Many studies have shown a link between smoking and increased risk of developing a breast carcinoma [5,6,7]. It has been proposed that tobacco smoke carcinogens pass through the alveolar membrane in the lungs and into the blood stream where they may be transported to the breast by plasma lipoproteins [8]. In vitro studies have proposed that cigarette smoke induces the epithelial to mesenchymal transition, producing a more aggressive phenotype [9]. A higher prevalence of tobacco metabolites and smoking-specific DNA adducts have been detected in the breast tissue of women who smoke than non-smokers [10,11]. Some studies have examined the risk of breast cancer according to genotypes that metabolize tobacco compounds in the human body or in genes related to DNA repair, with different results [3,12]. Genes such as *NAT1*, *NAT2*, *CYP1A1*, *COMT*, *BRCA1*, *BRCA2* or DNA repair genes have been reported to alter the relationship between smoking and breast cancer risk [7,13,14,15].

This case-control study was conducted to examine the association between active cigarette smoking (status, intensity, duration or age at initiation) and breast cancer risk among pre- and postmenopausal women and evaluate possible gene-environment interactions for selected SNPs in a cohort of Spanish women. Comparison of the associations of different smoking phenotypes with breast cancer risk may help not only to clarify the most relevant measures with regard to risk but also to determine the possible influence of smoking in various stages of breast cancer development.

## 2. Results

The study included 297 women, all of Caucasian (Spanish) descent for ≥3 generations, and the mean age was 54 years (SD = 13). Women were divided into three groups:

1. Seventy-six patients who had never smoked with histologically verified breast carcinoma, non-smoker group.
2. One hundred forty-one smoker patients with histologically verified breast carcinoma.
3. Eighty healthy smoker women, with negative family or personal history and with negative breast findings.

Table 1 provides a summary of demographic information, clinical parameters and active smoking variables (smoking status, age at initiation, PYS) and their average values in each group.

### 2.1. Smoking’s Impact on Clinical Variables and Breast Cancer

In the breast cancer group, dividing by smoking status, we observed statistically significant associations between smoking and diagnosis age (*p* < 0.001). In the non-smoker breast cancer group 19.7% were under 45 years old, while in the active smoker group it was 41.3% (*p* = 0.001). Moreover, among the smoker breast cancer group we also found a statically significant positive correlation between age at smoking initiation and diagnosis age of breast cancer (R = 0.294, *p* < 0.001).

Comparing breast cancer smokers versus healthy smokers, we observed statistically significant differences in age between two groups, but no statistically significant differences were observed in the age at smoking initiation or years smoking (*p* = 0.926; *p* = 0.154, respectively). On the other hand, the healthy smokers group smoked more CPD or presented higher PYS (*p* = 0.001; *p* = 0.002, respectively).

Among breast cancer patients, dividing by smoking status, we observed a non-significant different distribution of breast cancer subtypes (Table 1). Stratifying by YS (<20, ≥20), smoking duration (<20 years) was associated with significant increase in risk of in situ carcinoma (OR = 1.328, CI: 1.023–1.72). In addition, we found statistically significant associations between PYS (≤5, 5–15, ≥15) and different subtypes of breast cancer (*p* = 0.033). We observed a higher prevalence of invasive carcinoma in groups with more PYS (76.2%, 81.8% and 92.2%, respectively).

### 2.2. Analysis of Genetic Polymorphisms

We analysed the prevalence of the following genetic polymorphisms *XRCC1* (*Arg399Gln*) [rs25487], *APEX1* (*Asp148Glu*) [rs1130409], *XRCC3* (*Thr241Met*) [rs861539], *XPD* (*Asp312Asn*) [rs1799793] and (*Lys751Gln*) [rs13181], *CYP1A1* (*Ile462Val*) [rs1048943], CYP2A6*2 (*Lys160His*) [rs1801272], and CYP2A6*9 (−*48T > G*) [rs28399433] in breast cancer patients and healthy smokers.

No departure from the Hardy–Weinberg equilibrium was detected for the majority of genes, with the exception of *XRCC1* (*Arg399Gln*) (*p* = 0.002) and *XPD* (*Asp312Asn*) (*p* = 0.02).

Analyses focused on associations with genotype categorized using a recessive model (i.e., homozygotes of the most common allele plus heterozygotes were the referent group, compared to homozygotes of the minor allele).

### 2.3. Relationship between Smoking Habits, Clinical Variables and Genetic Polymorphisms

Among all smokers, no statistically relationship was observed between smoking habits or the clinical variables and genetic polymorphisms analysed. Only a moderately significant interaction was found between *XRCC3 Thr241allele* and more PYS (*p* = 0.039). This association was in agreement with previous reports [16]. In addition, we observed a marginal relationship between CYP2A6*2 and age of diagnosis in the group of smoker patients (*p* = 0.037).

### 2.4. Genetic Differences between Non-Smoker and Smoker Breast Cancer Patients

The frequencies of genetic polymorphisms studied were similar in both the smoker and non-smoker groups of patients (Table 2). We did not observe any interaction between the studied genetic polymorphisms and smoking in breast cancer distribution.

### 2.5. Genetic Differences between Smoker Controls and Smoker Breast Cancer Patients

Comparing only smoker groups, the frequencies of *XRCC1*, *APEX1*, *XPD*, CYP2A6*2 and CYP2A6*9 genetic polymorphisms were similar in both controls and breast cancer patients (Table 3). However, the *XRCC3* *Met241Met* genotype was associated with the risk of cancer development in smokers (*p* = 0.003, OR = 2.89 (1.35–6.21)). In contrast, the *CYP1A1* *Ile462Ile* genotype (most common genotype) was significantly associated with higher risk of breast cancer in smokers (*p* = 0.001, OR = 5.09 (1.94–13.36)).

The frequency of *CYP1A1 Ile462Val* was around 5% in breast cancer patients and 21% in healthy smokers, while the frequency of *XRCC3 Met241Met* was around 29% in breast cancer patients and 12% in healthy smokers, suggesting a possible interaction effect between *CYP1A1 Ile462Val*, *XRCC3* *Thr241Met* and risk of breast cancer in smokers, with regard to the risk for not developing breast cancer in smokers carrying the *CYP1A1 Ile462Val* genotype after interaction logistic regression analysis (*p* < 0.001, OR = 7.57 (2.48–23.25)).

Some studies have reported that duration of smoking is a much stronger risk factor than amount smoked [17,18,19,20]. Table 4 presents data of genetic frequencies for smokers stratified by smoking duration as a categorical variable (<20 YS/≥20 YS). Among smokers ≥20 YS, only *CYP1A1 Ile462Ile* was associated with the greater risk of breast cancer (OR = 7.12 (1.98–25.59)).

## 3. Discussion

Smoking represents a potential risk for breast cancer development. Tobacco smoke contains thousands of chemical compounds, many of which are known to be mammary carcinogens [21]. Cellular proliferation during the years of pre-puberty to age at first pregnancy is the highest and it may decrease the ability of DNA repair mechanisms to correct damage before cell division happens [22], as mammary tissue is more susceptible to carcinogenic exposures during this period [23]. We found that the association between smoking status and early age at diagnosis of breast cancer was significant. Moreover, our data suggested a younger age at smoking initiation is related to younger age at diagnosis. Smokers were diagnosed a mean of ten years before non-smokers. Smoking may accelerate the development of breast cancer and epidemiological findings suggest that smoking is associated with increased breast cancer incidence [6].

Among smokers, invasive carcinoma subtype incidence increased with the intensity and duration of smoking (all *P*_trend_ < 0.05). Such findings suggest that smoking can affect the incidence as well as the course of cancer. Di Cello and cols. have suggested that cigarette smoke promotes the epithelial to mesenchymal transition, producing a more aggressive breast cancer phenotype in vitro [9]. Susceptibility to breast cancer is a multifactorial trait (genetic components or environmental factors) that may differ between populations [24]. Certain genotypes of several genetic polymorphisms in enzymes involved in the metabolism of xenobiotics (such as CYP1A1) or in DNA repair genes have been suggested to alter the risk of breast cancer [25,26].

Among breast cancer group, we found the same genetic polymorphism prevalence to be independent of being a smoker or non-smoker. On the other hand, of the polymorphisms investigated comparing breast cancer smokers and healthy smokers, statistically significant associations between two genetic variants and risk of breast cancer in smokers were observed (*XRCC3* Met241, *CYP1A1* Ile462). However, the differences were the same independent of tobacco exposure. CYP1A1 metabolizes endogenous molecules and xenobiotics [27]. CYP1A1 catalyzes the first step in the metabolism of tobacco polycyclic aromatic hydrocarbons, leading to their carcinogenic activation [28]. CYP1A1 is also involved in the metabolism of estrogens, as one of the enzymes responsible for the 2-hydroxylation of 17h-estradiol and estrone in breast tissue [29,30]. Therefore, the activity of CYP1A1 may influence breast carcinogenesis via at least two distinct pathways, tobacco metabolites and estrogens.

These results may support the main effect of CYP1A1 in estrogenic metabolism rather than in tobacco carcinogen activation in breast cancer.

Our data also showed an interaction between *XRCC3 Thr241Met*, *CYP1A1 Ile462Val*. In addition, it has been suggested that lifetime smoking exposure, not just current situation, should be used to assess risk among smokers [31]. When smokers were stratified by smoking duration (YS), we only observed differences in the ≥20 YS group, and the *CYP1A1* *Ile462Ile* genotype was associated with increased risk of breast cancer. Smoking induces CYP1A1, and the effect of polymorphisms in estrogenic metabolism is upregulated in a dose-dependent manner [32].

Kisselev and cols. suggested that the effects of polymorphisms on the function of CYP1A1 may be dependent on the substrate, and CYP1A1 variants might differ from the wild type in terms of their activity and regioselectivity towards estrogens. From a biochemical point of view, the high estrogen-2-hydroxylase activity of the *CYP1A*1 *Ile562Val* variant may either increase or reduce the susceptibility to cancer depending on its combination with other genetic and environmental risk factors. The results of epidemiologic studies are controversial and obviously dependent on the ethnicity, gender, and lifestyle of the study group [33,34,35]. For example, an increased breast cancer risk associated with the CYP1A1*2 (*Ile462Val*) allele and smoking was observed in Caucasian women [35,36], whereas Chinese and Japanese women carrying the CYP1A1*2 (*Ile462Val*) allele have a significantly reduced risk of breast cancer as compared with carriers of the wild-type allele [37]. In addition, numerous studies have investigated the expression of *CYP1A1* in extra hepatic tissues which are largely exposed to environmental carcinogens. Expressional variation of *CYP1A1* has shown down-regulation of *CYP1A1* in breast cancer [38,39]. *CYP1A1 Ile462Val* substitution situated in the heme-binding region of the enzyme is accompanied by a twofold increase in microsomal enzyme activity [40]. Our results showed higher prevalence of *CYP1A1 562Val* in controls, and it is tempting to speculate that the CYP1A1*2 (*Ile462Val*) allele could even play a protective role in smokers.

In the literature, declared cigarette consumption often serves as a proxy measure for level of toxin exposure or disease risk. However, for different reasons, consumption may not be a good predictor. The differences in how tobacco is smoked affect real exposure, and it is difficult discern if consumption reported as cigarettes per day correlates with other markers of exposure, the effect of compensation (changes in smoking behaviour to adjust for changes in nicotine levels or in volume of cigarettes smoked), the complex relationship between dose and disease risk for some diseases or whether people maintain the same levels of smoking over time [41]. This is why at the beginning we decided to divide the breast cancer groups into smokers and never-smokers in order to clarify exposure levels.

In addition, based on more recent assessments with the assumption that the effect of the genotype might be amplified at high doses, we studied the effects of long exposure on breast cancer risk in heavy smokers (≥20 YS) [42]. We compared breast cancer smokers and healthy smokers stratified by YS and we replicated results found in all sample analysis.

The goal of our research was to combine environment (smoking history) with genetic polymorphisms to improve assessment risk of breast cancer. Moreover, we have included a group of patients who were never smokers to evaluate differences. Most previous studies of *CYP1A1* polymorphisms and breast cancer divided smokers into ever or never groups, and did not analyse the effects of intensity, years of smoking or age at initiation. Among the strengths of our study are the prospective design and selection of well-defined groups in terms of smoking. However, our study is subject to certain limitations such as low statistical power (because of low sample size) and passive smoking data not being included. There could be a “partial dose effect” from second hand smoke. We know that a weakness of our study is the low sample size, yet we believe this can be partly overcome by the fact that our population is homogeneous, not stratified and well defined in terms of phenotype assessment. These results suggest that cigarette smoking might play an important role in the development of breast cancer, particularly when women start smoking relatively early in life or when they are exposed for a long time. Our results confirm the hypothesis that *CYP1A1* *Ile462Val*, in association with a long period of active smoking, could be a possible breast cancer risk factor and also support the main effect of CYP1A1 in estrogenic metabolism rather than in tobacco carcinogen activation in breast cancer patients.

On the other hand, breast cancer is a complex disease that is mainly grouped based on its hormone receptor subtype (estrogen receptor or progesterone receptor positivity) and amplification of the *ERBB2* gene [43]. Evidence for smoking-related breast cancer risk in relation to hormone receptor status has been limited in Japan and Western countries [44,45]. Some studies have reported a positive association between ever smoking and the risk of estrogen receptor positive cancer [6,46,47], whereas others have reported a positive association for estrogen receptor negative cancer, or no association [47,48,49]. Further studies including bigger, well-phenotyped cohorts or hormone receptor subtype status would be useful to clarify the association of smoking with breast cancer risk.

## 4. Materials and Methods

### 4.1. Subject Enrolment and Data Collection

We designed a control-based study with 297 female patients in order to detect the possible association between smoking, polymorphisms in carcinogen metabolism and/or DNA repair genes, and risk of breast carcinoma development. Women were randomly recruited from Centro de Patología de la Mama (Fundación Tejerina), Madrid (Spain), from 2010 to 2013. The first group consisted of patients with histologically confirmed breast cancer (smokers and never smokers) and the second group consisted of healthy patients without a previous positive history of breast carcinoma or other malignancies and smokers with more than 1 pack years smoked (PYS). Approval was obtained on 4 November 2010 from the local Ethics Committee (2010/UEM19) and all patients provided written informed consent. The study was in accordance with the Helsinki Declaration.

All women completed a questionnaire regarding demographic characteristics, smoking habits, self-reported cigarettes per day (CPD), duration and intensity (PYS). CO expired and cotinine levels in urine samples were analyzed in 27% of smokers to check cigarette consumption. In addition, body mass index, tumour histology, type of treatment or concomitant medication data were collected for each participant.

### 4.2. Genotyping

Venous blood samples were collected by venepuncture and processed for leukocyte DNA extraction using a standard phenol chloroform protocol. DNA extraction and genotype analyses were carried out in the Biomedicine laboratory at the Universidad Europea, Madrid (Spain). The study followed recommendations for replicating genotype-phenotype association studies, genotyping was performed specifically for research purposes, and researchers in charge of genotyping were totally blinded to the participants’ identities (blood and DNA samples were tracked solely with bar-coding and personal identities were only made available to the main study researcher who was not involved in actual genotyping) [50]. The DNA samples were stored at −20 °C until analysis.

Genotyping was performed by real-time PCR and Taqman probes with a Step One Real-Time PCR System (Applied Biosystems, Foster City, CA, USA).

### 4.3. Statistical Analysis

Power analysis retrieved a sample size of 94 cases and 94 healthy patients necessary to detect an OR of 4 with a double-tale significant level of a = 0.05 and c2 = 0.80, assuming a prevalence of CYP1A1*2C allele of 6.4% in Caucasians [51].

We compared smoking phenotypes and clinical characteristics between the different groups with the unpaired Student’s *t*-test. We used the χ^2^ test to assess deviations of genotype distributions from the Hardy-Weinberg equilibrium (HWE). We also used logistic regression to determine associations between smoking habits, breast cancer risk and genotypes. All statistical analyses were corrected for multiple comparisons using the Bonferroni method, in which the threshold *p*-value is obtained by dividing 0.05 by the number of tests. The data were stratified for smoking status, smoking duration or smoking intensity to determine several associations. All analyses were performed with the PASW/SPSS Statistics 20.0 (SPSS Inc., Chicago, IL, USA) program.

## 5. Conclusions

Cigarette smoking might play an important role in the development of breast cancer, particularly when women start smoking relatively early in life or when they are exposed for a long time. Larger studies are needed to confirm our initial findings, however, our results provide evidence that the main effect of CYP1A1 in estrogenic metabolism rather than in tobacco carcinogen activation in breast cancer patients and also confirmed the hypothesis that CYP1A1 Ile462Val, in association with long periods of active smoking, could be a breast cancer risk factor.

## Figures and Tables

**Table 1:** Summary for demographic, clinical and tobacco consumption variables among the population.

Variables		Breast Carcinoma Group		Healthy Group
		Non-Smokers	Smokers	Smokers
Age (years)		64 ± 10	56 ± 9	42 ± 11
Age at diagnosis (years)		56 ± 11	48 ± 9	-
Breast cancer before age 45 years (%)		19.7%	41.3%	-
Smoking duration (years)		-	25.5 ± 11.4	22.9 ± 11.8
Age at initiation (years)		-	18 ± 5	18 ± 5
CPD		-	14.0 ± 8.7	19.3 ± 13.1
PYS		-	17.2 ± 13.8	25.5 ± 27.4
Heavy smokers 15 ≥ PYS (%)		-	47.9%	55.0%
Breast cancer histology	In situ carcinoma (%)	22.9%	14.6%	-
	Invasive (infiltrating carcinoma) (%)	77.1%	85.4%	-

The values are given as mean ± SD. Abbreviations: CPD, cigarettes per day; PYS, pack years smoked.

**Table 2:** Genotypes between breast cancer groups stratified by smoking.

Genotype	Breast Cancer Patients			
	Non-Smokers	Smokers	*p*	OR (95% CI)
***XRCC1* Arg399Gln**				
Arg/Arg, Arg/Gln ^a^	78.3	75.7	0.413	1.15 (0.57–2.31)
Gln/Gln	21.7	24.3		
***APEX1* Asp148Glu**				
Asp/Asp, Asp/Glu ^a^	75.0	67.9	0.192	0.78 (0.48–1.26)
Glu/Glu	25.0	32.1		
***XRCC3* Thr241Met**				
Thr/Thr, Thr/Mer ^a^	70.4	71.0	0.528	1.02 (0.65–1.59)
Met/Met	29.6	29.0		
***ERCC2* Asp312Asn**				
Asp/Asp, Asp/Asn ^a^	85.9	85.6	0.565	0.97 (0.48–1.99)
Asn/Asn	14.1	14.4		
***KLC3* Lys751Gln**				
Lys/Lys, Lys/Gln ^a^	83.6	81.7	0.452	0.89 (0.47–1.72)
Gln/Gln	16.4	18.3		
***CYP1A1* Ile462Val**				
Ile/Ile ^a^	95.9	94.9	0.511	0.79 (0.21–2.98)
Ile/Val	4.1	5.1		
***CYP2A6* Lys160His**				
Lys/Lys	100	93	0.039	1.07 (1.02–1.13)
Lys/His ^a^	0	7		
***CYP2A6 –*48T > G**				
TT/TG ^a^	87	85.5	0.494	0.89 (0.39–2.03)
GG	13	14.3		

^a^ Reference genotype; OR: overall risk for non-smokers; CI: confidence interval.

**Table 3:** Association between genotype and risk of breast cancer among smokers.

Genotype	Smokers Group			
	Breast Cancer Group	Healthy Group	*p*	OR (95% CI)
***XRCC1* Arg399Gln**				
Arg/Arg, Arg/Gln ^a^	75.7	82.3	0.172	1.37 (0.78–2.40)
Gln/Gln	24.3	17.7		
***APEX1* Asp148Glu**				
Asp/Asp, Asp/Glu ^a^	67.9	66.7	0.483	0.96 (0.64–1.44)
Glu/Glu	32.1	33.3		
***XRCC3* Thr241Met**				
Thr/Thr, Thr/Mer ^a^	71.0	87.7	**0.003**	2.89 (1.35–6.21)
Met/Met	29.0	12.3		
***ERCC2* Asp312Asn**				
Asp/Asp, Asp/Asn ^a^	85.6	87.5	0.432	1.15 (0.56–2.35)
Asn/Asn	14.4	12.5		
***KLC3* Lys751Gln**				
Lys/Lys, Lys/Gln ^a^	81.7	86.2	0.283	1.32 (0.65–2.68)
Gln/Gln	18.3	13.8		
***CYP1A1* Ile462Val**				
Ile/Ile	94.9	78.5	**0.001**	5.09 (1.94–13.36)
Ile/Val ^a^	5.1	21.5		
***CYP2A6* Lys160His**				
Lys/Lys ^a^	93	91.4	0.460	0.81 (0.28–2.37)
Lys/His	7	8.6		
***CYP2A6 –*48T>G**				
TT/TG ^a^	85.5	83.3	0.437	0.87 (0.42–1.80)
GG	14.3	16.7		

^a^ Reference genotype; OR: overall risk for breast cancer; CI: confidence interval. The bold numbers in the table show statistically significant (*p* < 0.006).

**Table 4:** Genotypes among smoker groups stratified by smoking duration.

Genotype	Years Smoking < 20				Years Smoking ≥ 20			
	Breast Cancer Group	Healthy Group	*p*	OR (95% CI)	Breast Cancer Group	Healthy Group	*p*	OR (95% CI)
***XRCC1* Arg399Gln**								
Arg/Arg, Arg/Gln ^a^	65.0	87.9	0.022	2.88 (1.05–7.93)	80.4	77.8	0.440	0.88 (0.44–1.74)
Gln/Gln	35.0	12.1			19.6	22.2		
***APEX1* Asp148Glu**								
Asp/Asp, Asp/Glu ^a^	74.4	78.1	0.466	1.17 (0.50–2.73)	62.5	57.8	0.258	0.82 (0.53–1.29)
Glu/Glu	25.6	21.9			34.8	42.2		
***XRCC3* Thr241Met**								
Thr/Thr, Thr/Mer ^a^	69.2	87.9	0.052	2.5 (0.90–7.13)	73.0	89.4	0.020	2.53 (1.03–6.21)
Met/Met	30.8	12.1			27.0	10.6		
***ERCC2* Asp312Asn**								
Asp/Asp, Asp/Asn ^a^	86.8	84.8	0.538	0.87 (0.27–2.74)	85.6	89.1	0.385	1.32 (0.50–3.50)
Asn/Asn	13.2	15.2			14.4	10.9		
***KLC3* Lys751Gln**								
Lys/Lys, Lys/Gln ^a^	82.1	78.6	0.479	0.83 (0.31–2.22)	81.8	91.7	0.132	2.18 (0.67–7.03)
Gln/Gln	17.9	21.4			18.2	8.3		
***CYP1A1* Ile462Val**								
Ile/Ile	92.5	83.9	0.222	1.10 (0.92–1.32)	95.7	75.8	**0.002**	7.12 (1.98–25.59)
Ile/Val ^a^	7.5	16.1			4.3	24.2		
***CYP2A6* Lys160His**								
Lys/Lys ^a^	96.9	90.0	0.282	0.31 (0.03–2.84)	92.4	92.6	0.670	1.02 (0.22–4.78)
Lys/His	3.1	10.0			7.6	7.4		
***CYP2A6−*48T > G**								
TT/TG ^a^	84.4	87.5	0.500	1.25 (0.37–4.23)	86.7	77.8	0.214	0.60 (0.24–1.49)
GG	15.6	12.5			13.3	22.2		

^a^ Reference genotype; OR: overall risk for breast cancer; CI: confidence interval. The bold number in the table shows statistically significant (*p* < 0.006).

## References

[1] World Health Organization (2011). Anonymous WHO urges more countries to require large, graphic health warnings on tobacco packaging: The WHO report on the global tobacco epidemic, 2011 examines anti-tobacco mass-media campaigns. Cent. Eur. J. Public Health.

[2] Reynolds P. (2013). Smoking and breast cancer. J. Mammary Gland Biol. Neoplasia.

[3] Terry P.D., Goodman M. (2006). Is the association between cigarette smoking and breast cancer modified by genotype? A review of epidemiologic studies and meta-analysis. Cancer Epidemiol. Biomark. Prev..

[4] Young E., Leatherdale S., Sloan M., Kreiger N., Barisic A. (2009). Age of smoking initiation and risk of breast cancer in a sample of Ontario women. Tob. Induc. Dis..

[5] Stephenson N., Beckmann L., Chang-Claude J. (2010). Carcinogen metabolism, cigarette smoking, and breast cancer risk: A Bayes model averaging approach. Epidemiol. Perspect. Innov..

[6] Gaudet M.M., Gapstur S.M., Sun J., Diver W.R., Hannan L.M., Thun M.J. (2013). Active smoking and breast cancer risk: Original cohort data and meta-analysis. J. Natl. Cancer Inst..

[7] Kasajova P., Holubekova V., Mendelova A., Lasabova Z., Zubor P., Kudela E., Biskupska-Bodova K., Danko J. (2016). Active cigarette smoking and the risk of breast cancer at the level of *N*-acetyltransferase 2 (NAT2) gene polymorphisms. Tumour Biol..

[8] Plant A.L., Benson D.M., Smith L.C. (1985). Cellular uptake and intracellular localization of benzo(a)pyrene by digital fluorescence imaging microscopy. J. Cell Biol..

[9] Di Cello F., Flowers V.L., Li H., Vecchio-Pagan B., Gordon B., Harbom K., Shin J., Beaty R., Wang W., Brayton C. (2013). Cigarette smoke induces epithelial to mesenchymal transition and increases the metastatic ability of breast cancer cells. Mol. Cancer.

[10] Petrakis N.L., Gruenke L.D., Beelen T.C., Castagnoli N., Craig J.C. (1978). Nicotine in breast fluid of nonlactating women. Science.

[11] Rundle A., Tang D., Zhou J., Cho S., Perera F. (2000). The association between glutathione S-transferase M1 genotype and polycyclic aromatic hydrocarbon-DNA adducts in breast tissue. Cancer Epidemiol. Biomarkers Prev..

[12] Anderson L.N., Cotterchio M., Mirea L., Ozcelik H., Kreiger N. (2012). Passive cigarette smoke exposure during various periods of life, genetic variants, and breast cancer risk among never smokers. Am. J. Epidemiol..

[13] Andres S.A., Bickett K.E., Alatoum M.A., Kalbfleisch T.S., Brock G.N., Wittliff J.L. (2015). Interaction between smoking history and gene expression levels impacts survival of breast cancer patients. Breast Cancer Res. Treat..

[14] Cotterchio M., Mirea L., Ozcelik H., Kreiger N. (2014). Active cigarette smoking, variants in carcinogen metabolism genes and breast cancer risk among pre- and post-menopausal women in Ontario, Canada. Breast J..

[15] Cox D.G., Dostal L., Hunter D.J., le Marchand L., Hoover R., Ziegler R.G., Thun M.J. (2011). Breast and Prostate Cancer Cohort Consortium: *N*-acetyltransferase 2 polymorphisms, tobacco smoking, and breast cancer risk in the breast and prostate cancer cohort consortium. Am. J. Epidemiol..

[16] Verde Z., Reinoso L., Chicharro L.M., Resano P., Sanchez-Hernandez I., Rodriguez Gonzalez-Moro J.M., Bandres F., Gomez-Gallego F., Santiago C. (2015). Are SNP-Smoking Association Studies Needed in Controls? DNA Repair Gene Polymorphisms and Smoking Intensity. PLoS ONE.

[17] Doll R., Peto R. (1978). Cigarette smoking and bronchial carcinoma: Dose and time relationships among regular smokers and lifelong non-smokers. J. Epidemiol. Community Health.

[18] Godtfredsen N.S., Holst C., Prescott E., Vestbo J., Osler M. (2002). Smoking reduction, smoking cessation, and mortality: A 16-year follow-up of 19,732 men and women from The Copenhagen Centre for Prospective Population Studies. Am. J. Epidemiol..

[19] Husten C.G. (2009). How should we define light or intermittent smoking? Does it matter?. Nicotine Tob. Res..

[20] Park S.J., Yi B., Lee H.S., Oh W.Y., Na H.K., Lee M., Yang M. (2016). To quit or not: Vulnerability of women to smoking tobacco. J. Environ. Sci. Health C Environ. Carcinog. Ecotoxicol. Rev..

[21] Hecht S.S. (2002). Cigarette smoking and lung cancer: Chemical mechanisms and approaches to prevention. Lancet Oncol..

[22] Russo A.L., Thiagalingam A., Pan H., Califano J., Cheng K.H., Ponte J.F., Chinnappan D., Nemani P., Sidransky D., Thiagalingam S. (2005). Differential DNA hypermethylation of critical genes mediates the stage-specific tobacco smoke-induced neoplastic progression of lung cancer. Clin. Cancer Res..

[23] Marcus P.M., Hayes R.B., Vineis P., Garcia-Closas M., Caporaso N.E., Autrup H., Branch R.A., Brockmoller J., Ishizaki T., Karakaya A.E. (2000). Cigarette smoking, *N*-acetyltransferase 2 acetylation status, and bladder cancer risk: A case-series meta-analysis of a gene-environment interaction. Cancer Epidemiol. Biomark. Prev..

[24] Li D., Walcott F.L., Chang P., Zhang W., Zhu J., Petrulis E., Singletary S.E., Sahin A.A., Bondy M.L. (2002). Genetic and environmental determinants on tissue response to in vitro carcinogen exposure and risk of breast cancer. Cancer Res..

[25] Ali K., Mahjabeen I., Sabir M., Mehmood H., Kayani M.A. (2015). OGG1 Mutations and Risk of Female Breast Cancer: Meta-Analysis and Experimental Data. Dis. Markers.

[26] Tyndale R.F., Sellers E.M. (2002). Genetic variation in CYP2A6-mediated nicotine metabolism alters smoking behavior. Ther. Drug Monit..

[27] Nebert D.W., Wikvall K., Miller W.L. (2013). Human cytochromes P450 in health and disease. Philos. Trans. R Soc. Lond. B Biol. Sci..

[28] McManus M.E., Burgess W.M., Veronese M.E., Huggett A., Quattrochi L.C., Tukey R.H. (1990). Metabolism of 2-acetylaminofluorene and benzo(a)pyrene and activation of food-derived heterocyclic amine mutagens by human cytochromes P-450. Cancer Res..

[29] Lee A.J., Cai M.X., Thomas P.E., Conney A.H., Zhu B.T. (2003). Characterization of the oxidative metabolites of 17β-estradiol and estrone formed by 15 selectively expressed human cytochrome p450 isoforms. Endocrinology.

[30] Monostory K., Dvorak Z. (2011). Steroid regulation of drug-metabolizing cytochromes P450. Curr. Drug Metab..

[31] Saquib N., Stefanick M.L., Natarajan L., Pierce J.P. (2013). Mortality risk in former smokers with breast cancer: Pack-years vs. smoking status. Int. J. Cancer.

[32] Shizu M., Itoh Y., Sunahara R., Chujo S., Hayashi H., Ide Y., Takii T., Koshiko M., Chung S.W., Hayakawa K. (2008). Cigarette smoke condensate upregulates the gene and protein expression of proinflammatory cytokines in human fibroblast-like synoviocyte line. J. Interferon Cytokine Res..

[33] Mitrunen K., Hirvonen A. (2003). Molecular epidemiology of sporadic breast cancer. The role of polymorphic genes involved in oestrogen biosynthesis and metabolism. Mutat. Res..

[34] Miyoshi Y., Noguchi S. (2003). Polymorphisms of estrogen synthesizing and metabolizing genes and breast cancer risk in Japanese women. Biomed. Pharmacother..

[35] Ambrosone C.B., Freudenheim J.L., Marshall J.R., Graham S., Vena J.E., Brasure J.R., Michalek A.M., Laughlin R., Nemoto T., Shields P.G. (1995). The association of polymorphic *N*-acetyltransferase (NAT2) with breast cancer risk. Ann. N. Y. Acad. Sci..

[36] Ishibe N., Hankinson S.E., Colditz G.A., Spiegelman D., Willett W.C., Speizer F.E., Kelsey K.T., Hunter D.J. (1998). Cigarette smoking, cytochrome P450 1A1 polymorphisms, and breast cancer risk in the Nurses’ Health Study. Cancer Res..

[37] Miyoshi Y., Takahashi Y., Egawa C., Noguchi S. (2002). Breast cancer risk associated with CYP1A1 genetic polymorphisms in Japanese women. Breast J..

[38] Bernauer U., Heinrich-Hirsch B., Tonnies M., Peter-Matthias W., Gundert-Remy U. (2006). Characterisation of the xenobiotic-metabolizing Cytochrome P450 expression pattern in human lung tissue by immunochemical and activity determination. Toxicol. Lett..

[39] Hafeez S., Ahmed A., Rashid A.Z., Kayani M.A. (2012). Down-regulation of CYP1A1 expression in breast cancer. Asian Pac. J. Cancer Prev..

[40] Kisselev P., Schunck W.H., Roots I., Schwarz D. (2005). Association of CYP1A1 polymorphisms with differential metabolic activation of 17β-estradiol and estrone. Cancer Res..

[41] Verde Z., Reinoso-Barbero L., Chicharro L., Garatachea N., Resano P., Sanchez-Hernandez I., Gonzalez-Moro J.M.R., Bandres F., Santiago C., Gomez-Gallego F. (2015). Effects of cigarette smoking and nicotine metabolite ratio on leukocyte telomere length. Environ. Res..

[42] Cox L.A. (2012). Low-dose nonlinear effects of smoking on coronary heart disease risk. Dose. Response.

[43] Sorlie T., Perou C.M., Tibshirani R., Aas T., Geisler S., Johnsen H., Hastie T., Eisen M.B., van de Rijn M., Jeffrey S.S. (2001). Gene expression patterns of breast carcinomas distinguish tumor subclasses with clinical implications. Proc. Natl. Acad. Sci. USA.

[44] Terry P.D., Rohan T.E. (2002). Cigarette smoking and the risk of breast cancer in women: A review of the literature. Cancer Epidemiol. Biomark. Prev..

[45] Althuis M.D., Fergenbaum J.H., Garcia-Closas M., Brinton L.A., Madigan M.P., Sherman M.E. (2004). Etiology of hormone receptor-defined breast cancer: A systematic review of the literature. Cancer Epidemiol. Biomark. Prev..

[46] Yoo K.Y., Tajima K., Miura S., Takeuchi T., Hirose K., Risch H., Dubrow R. (1997). Breast cancer risk factors according to combined estrogen and progesterone receptor status: A case-control analysis. Am. J. Epidemiol..

[47] Hunter D.J., Hankinson S.E., Hough H., Gertig D.M., Garcia-Closas M., Spiegelman D., Manson J.E., Colditz G.A., Willett W.C., Speizer F.E. (1997). A prospective study of NAT2 acetylation genotype, cigarette smoking and risk of breast cancer. Carcinogenesis.

[48] Cooper J.A., Rohan T.E., Cant E.L., Horsfall D.J., Tilley W.D. (1989). Risk factors for breast cancer by oestrogen receptor status: A population-based case-control study. Br. J. Cancer.

[49] Nishino Y., Minami Y., Kawai M., Fukamachi K., Sato I., Ohuchi N., Kakugawa Y. (2014). Cigarette smoking and breast cancer risk in relation to joint estrogen and progesterone receptor status: A case-control study in Japan. Springerplus.

[50] Chanock S.J., Manolio T., Boehnke M., Boerwinkle E., Hunter D.J., Thomas G., Hirschhorn J.N., Abecasis G., Altshuler D., NCI-NHGRI Working Group on Replication in Association Studies (2007). Replicating genotype-phenotype associations. Nature.

[51] Garte S., Gaspari L., Alexandrie A.K., Ambrosone C., Autrup H., Autrup J.L., Baranova H., Bathum L., Benhamou S., Boffetta P. (2001). Metabolic gene polymorphism frequencies in control populations. Cancer Epidemiol. Biomark. Prev..

